# Supplementary material for: Biological Evaluation of 8-Methoxy-2,5-dimethyl-5H-indolo[2,3-b] Quinoline as a Potential Antitumor Agent via PI3K/AKT/mTOR Signaling
Source: Int J Mol Sci. 2023 Oct 13;24(20):15142. doi: 10.3390/ijms242015142 (PMC10606936; doi:10.3390/ijms242015142)
Supplement: Supplementary file 1 [file ijms-24-15142-s001.zip › ijms-2621538-supplementary.pdf]

Supporting Materials

Biological evaluation of the neocryptolepine derivative MMNC as a potential antitumor agent by PI3K/AKT/mTOR signaling

Yunhao Ma[1, †], Xinrong Jiang[1, †], Zhongkun Zhou[1], Yong Zhou[1], Yanan Tian[2], Hao Zhang[1], Mengze Sun[1], Lixue Tu[1], Juan Lu[1], Yuqing Niu[1], Huanxiang Liu[2], Hongmei Zhu[1, *], Yingqian Liu[1, *], Peng Chen[1, *]

[1] School of Pharmacy, Lanzhou University, No. 199 Donggang West Road, Lanzhou, 730000, People's Republic of China

[2] Faculty of Applied Science, Macao Polytechnic University, Macao, China

---

† The authors contributed equally to this work.

* Correspondence and requests for materials should be addressed to Hongmei Zhu (E-mail: hmzhu@lzu.edu.cn) or Yingqian Liu (E-mail: yqliu@lzu.edu.cn) or Peng Chen (E-mail: chenpeng@lzu.edu.cn). Tel. & fax: +86 931 8915686.

Table S1 The molecular docking scoring of MMNC and proteins

Figure S1 ^{1}H NMR spectra of MMNC

Figure S2 ^{13}C NMR spectra of MMNC

Figure S3 Mass spectrometry of MMNC

Figure S4 Purity test of MMNC by HPLC

Table S1. The molecular docking scoring of MMNC and proteins

Target	PDB ID	Resolution (Å)	Organisms	Docking score
CDK1	4y72	2.3	*Homo sapiens*	-8.834
MEK	5eym	2.7	*Homo sapiens*	-7.613
PI3K	6gvf	2.5	*Homo sapiens*	-7.543
mTOR	4jt6	3.6	*Homo sapiens*	-7.45
β-catenin	3tx7	2.76	*Homo sapiens*	-7.397
JNK	4y5h	2.06	*Homo sapiens*	-7.207
P21	5bms	2.9	*Homo sapiens*	-7.137
Raf	5fd2	2.89	*Homo sapiens*	-7.057
Cyclin E_1	5l2w	2.8	*Homo sapiens*	-6.963
Cyclin A	6gue	1.99	*Homo sapiens*	-6.793
AKT	6hhf	2.9	*Homo sapiens*	-6.436
MAPK	6tca	3.7	*Homo sapiens*	-6.113
JAK	5tq6	2.06	*Homo sapiens*	-5.91
Caspase-3	6x8k	2.17	*Homo sapiens*	-5.536
E-cadherin	3l6y	3	*Homo sapiens*	-4.314
CDK6	1jow	3.1	*Homo sapiens*	-4.105
Ras	7jhp	2.77	*Homo sapiens*	-4.048
Bcl-2	4b4s	1.9	*Homo sapiens*	-3.987

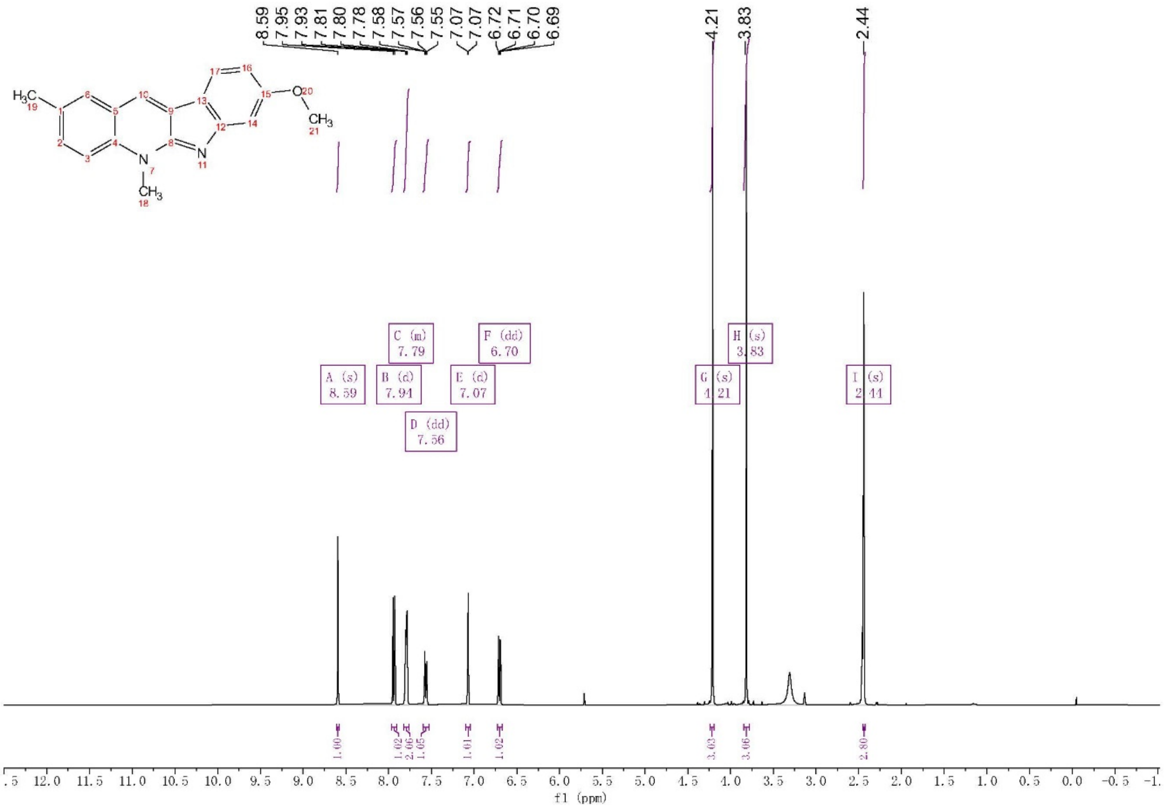

Figure S1 ^{1}H NMR spectra of MMNC

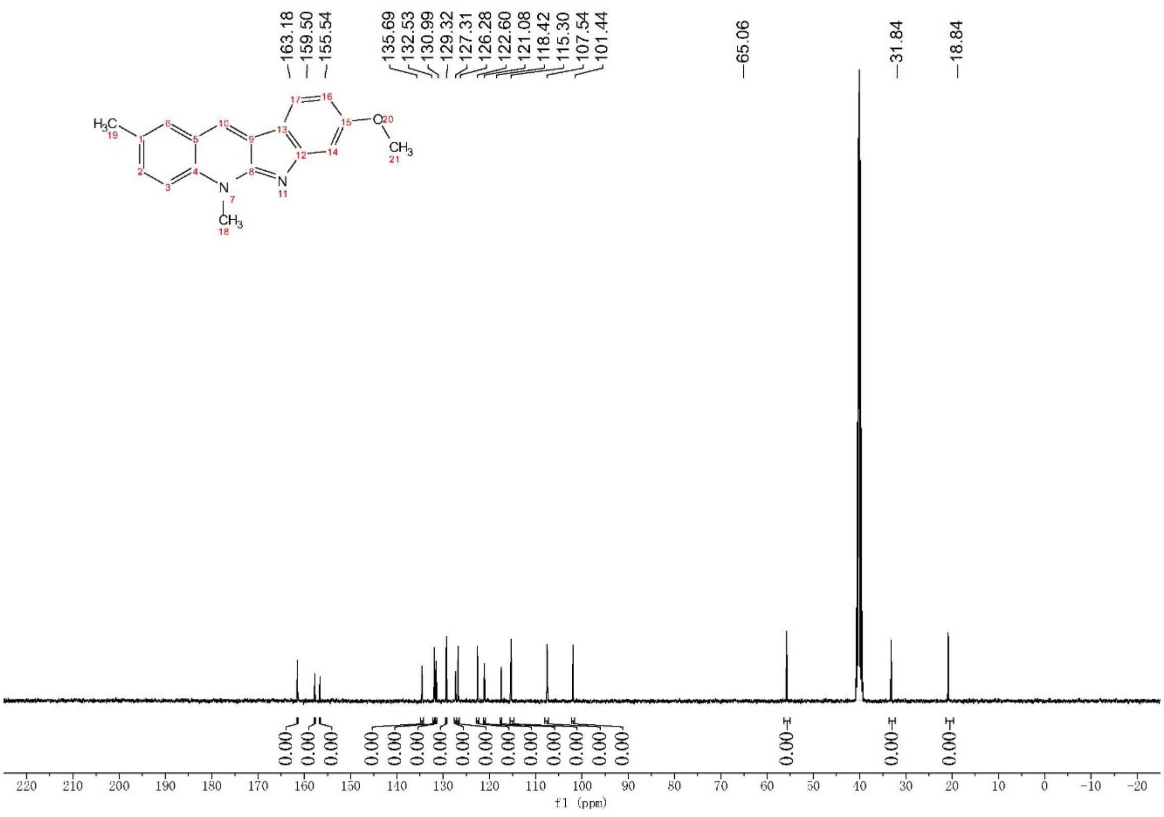

Figure S2 ^{13}C NMR spectra of MMNC

**Sample Name**		**Position**		**Instrument Name**	
**User Name**		**Inj Vol**		**InjPosition**	
**Sample Type**		**IRM Calibration Status**	Success	**Data Filename**	20220905-jiangxinrong-Z44-002.d
**ACQ Method**		**Comment**		**Acquired Time**	

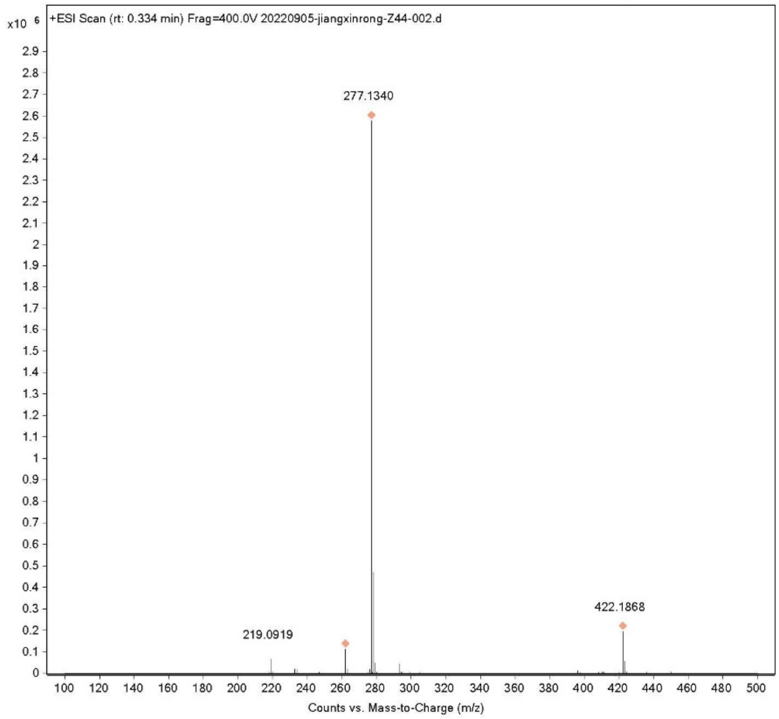

Figure S3 Mass spectrometry of MMNC

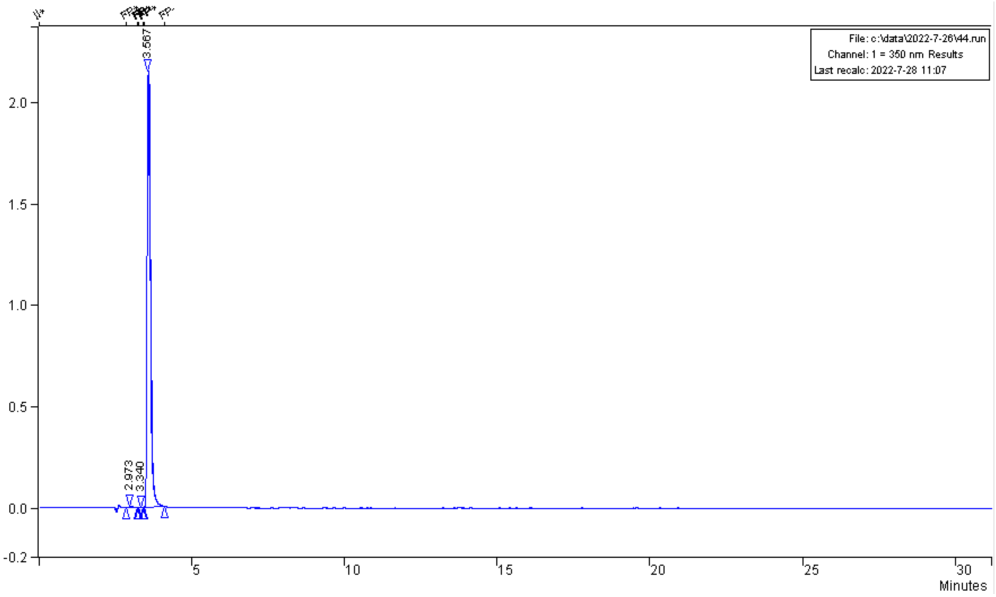

Figure S4 Purity test of MMNC by HPLC
